# Enhanced Charge Collection in MOF‐525–PEDOT Nanotube Composites Enable Highly Sensitive Biosensing

**DOI:** 10.1002/advs.201700261

**Published:** 2017-09-22

**Authors:** Tzu‐Yen Huang, Chung‐Wei Kung, Yu‐Te Liao, Sheng‐Yuan Kao, Mingshan Cheng, Ting‐Hsiang Chang, Joel Henzie, Hatem R. Alamri, Zeid A. Alothman, Yusuke Yamauchi, Kuo‐Chuan Ho, Kevin C.‐W. Wu

**Affiliations:** ^1^ Department of Chemical Engineering National Taiwan University No. 1, Sec. 4, Roosevelt Road Taipei 10617 Taiwan; ^2^ International Center for Materials Nanoarchitectonics (MANA) National Institute for Materials Science (NIMS) 1‐1 Namiki Tsukuba Ibaraki 305‐0044 Japan; ^3^ Physics Department Jamoum University College Umm Al‐Qura University Makkah 21955 Saudi Arabia; ^4^ Advanced Materials Research Chair Chemistry Department College of Science King Saud University Riyadh 11451 Saudi Arabia; ^5^ Division of Medical Engineering Research National Health Research Institute Keyan Road Zhunan Miaoli City 350 Taiwan

**Keywords:** biosensing, dopamine, metal‐organic frameworks, nanotubes, PEDOT, PC12 cells

## Abstract

With the aim of a reliable biosensing exhibiting enhanced sensitivity and selectivity, this study demonstrates a dopamine (DA) sensor composed of conductive poly(3,4‐ethylenedioxythiophene) nanotubes (PEDOT NTs) conformally coated with porphyrin‐based metal–organic framework nanocrystals (MOF‐525). The MOF‐525 serves as an electrocatalytic surface, while the PEDOT NTs act as a charge collector to rapidly transport the electron from MOF nanocrystals. Bundles of these particles form a conductive interpenetrating network film that together: (i) improves charge transport pathways between the MOF‐525 regions and (ii) increases the electrochemical active sites of the film. The electrocatalytic response is measured by cyclic voltammetry and differential pulse voltammetry techniques, where the linear concentration range of DA detection is estimated to be 2 × 10^−6^–270 × 10^−6^ m and the detection limit is estimated to be 0.04 × 10^−6^ m with high selectivity toward DA. Additionally, a real‐time determination of DA released from living rat pheochromocytoma cells is realized. The combination of MOF5‐25 and PEDOT NTs creates a new generation of porous electrodes for highly efficient electrochemical biosensing.

## 1 Introduction

Dopamine (DA) is an important biochemical messenger that plays a critical role in transmitting signals in the nervous system.^1^ Imbalances in DA cause various neurodegenerative diseases such as Parkinson's disease.^2^ Hence, analytical techniques that can accurately access levels of DA in the body would lead to better outcomes in medical investigations. Numerous methods for DA detection exist including chemiluminescence,^3^ electrochemiluminescence,^4^ fast‐scan cyclic voltammetry (FSCV),^5^ and fast fluorescence spectroscopy.^6^ While each method has some advantages, direct electrochemical detection methods offer a reliable, low‐cost approach with high sensitivity and selectivity for fast detection of DA.^7^ Additionally, label‐free electrochemical monitoring of DA released from living cells would enable rapid clinical diagnosis and potentially help mitigate or even prevent neuronal disorders and disease.

Metal‐organic frameworks (MOFs) consist of organic molecular linkers bonded to metal‐based nodes.^8^ MOFs have advantages such as tunable porosity, chemical stability, ultrahigh specific surface area, and ability to tune the surface chemistry. These features have enabled MOFs to find applications in diverse research fields including heterogeneous catalysis,^9^ gas storage,^10^ separation,^11^ capture,^12^ and chemical sensing.^13^ The electrochemical properties of MOFs have recently received significant attention in the chemical literatures.^14^ MOFs constructed with porphyrin subunits are particularly interesting because of their redox activity and have been tested as electrodes to detect organohalide pollutants,^15^ oxygen,^16^ and thrombin.^17^ The MOF architecture is important because these porphyrin‐based materials such as MOF‐525 have high specific surface areas, and the electrochemical activity of the porphyrin subunit can be tuned for different electrochemical sensing applications.^18^ Yet these porphyrin‐based MOFs still have limited sensitivity in electrochemical assays because individual MOF regions suffer from slow charge transport. An integration of porphyrin‐based MOFs into a conducting polymer would be a solution. Based on our previous experience on MOFs and conductive polymers,^19^ we propose to synthesize hybrid nanocomposites composed of a conductive polymer poly(3,4‐ethylenedioxythiophene) with a tubular morphology (namely, PEDOT NTs) conformally coated with porphyrin‐based MOF‐525 nanocrystals.

These pioneering studies of porphyrin ‐based MOF and PEDOT nanotubes inspire us to combine the advantages of these two materials for effective biosensing of DA. In this study, we describe an in situ method to synthesize hybrid nanocomposites composed PEDOT nanotubes conformally coated with porphyrin‐based MOF‐525 nanocrystals. The MOF‐525 nanocrystals function as electrode materials with numerous electrochemically active sites, while the PEDOT NTs serve as charge collectors to efficiently transport electrons to the electrode. Combining them to make MOF‐525–PEDOT NTs nanocomposite structures provide synergistic effects that result in a marked improvement in conductivity and catalytic performance, enhancing their ability to unequivocally sense DA with a good linear concentration range and detection of limit. In addition, we applied rat pheochromocytoma (PC12) cell line for DA detection because PC12 cells possess similar characteristics to that of mature sympathetic neurons. Furthermore, these composite films can directly measure the DA released from living PC12 cells, establishing this system as a practical platform for reliable and robust chemical sensing.

## 2 Results and Discussion

### 2.1 Characterization of MOF‐525–PEDOT NTs Composite Materials

**Figure 1:**
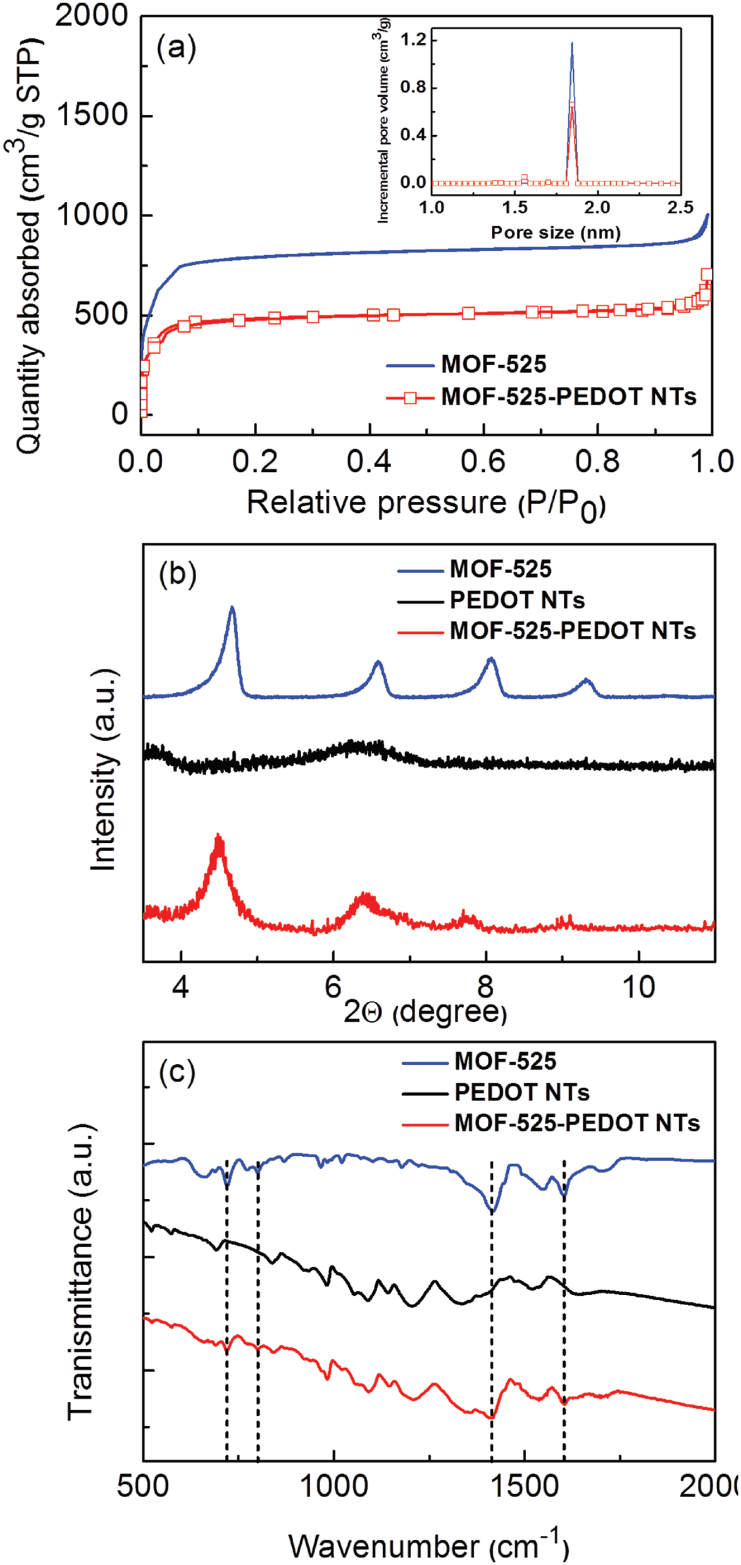
a) Nitrogen adsorption–desorption isotherms of MOF‐525 nanocrystals and MOF‐525–PEDOT NTs nanocomposite. b) and c) The XRD pattern and IR spectra of MOF‐525, PEDOT NTs, and MOF‐525–PEDOT NTs nanocomposite.

High porosity and large surface area are the most distinguishing features of MOF materials, thus we characterized MOF‐525 nanocrystals and MOF‐525–PEDOT NTs nanocomposites with gas absorption. The N2 adsorption–desorption curves of MOF‐525 nanocrystals and MOF‐525–PEDOT NTs nanocomposite are shown in **Figure** **1**a. The MOF‐525 nanocrystals show a Brunauer–Emmett–Teller (BET) specific surface area of 2690 m^2^ g^−1^, which is similar to other works.^20^, ^21^ Incorporating PEDOT caused the BET specific surface area of MOF‐525–PEDOT NTs to decrease to 1650 m^2^ g^−1^. Both curves had similar microporous adsorption–desorption behavior, indicating that the MOF‐525–PEDOT NT nanocomposites maintain the characteristic porous properties of the parent MOF‐525 nanocrystals. The MOF‐525 had a pore size of 1.84 nm as calculated by the density functional theory method using a cylindrical‐pore model (Figure 1a; inset).^20^, ^21^ The MOF‐525–PEDOT NT nanocomposites exhibited a similar pore size, showing that the nanocomposites contain the pure, highly crystalline MOF‐525 nanocrystals. In Figure 1b, the crystal structures of pristine MOF‐525, PEDOT NTs, and MOF‐525–PEDOT NTs nanocomposite were characterized by X‐ray diffractometer (XRD). Diffraction peaks of MOF‐525 nanocrystals appear at 2θ = 4.6°, 6.5°, 8.0°, and 9.2°; these peaks agree well with the previously reported XRD pattern,^21^ whereas the XRD pattern of PEDOT NTs showed no obvious peaks owing to the amorphous structure.^22^ The MOF‐525–PEDOT NT nanocomposite had diffraction peaks at 2θ = 4.5°, 6.4°, and 7.8°, indicating the same characteristic structure of MOF‐525.

The functional groups of the samples were examined with Fourier‐transform infrared (FT‐IR) spectroscopy. As shown in Figure 1c, the FT‐IR spectrum of the MOF‐525 nanocrystals had the characteristic bands (1705, 1606, 1416, 1178, 1003, 872, 800, 775, and 721 cm^−1^) that were previously reported.^23^ For PEDOT NTs, the peaks at 690, 839, and 981 cm^−1^ are related to C—S bond stretching vibration of the thiophene rings, and the peaks at 1344 and 1518 cm^−1^ correspond to C—C or C=C stretching of thiophene rings. The peaks at 1052, 1089, and 1204 cm^−1^ could be attributed to stretching of the C—O—C bonds. The MOF‐525–PEDOT NTs composites possess a similar FT‐IR spectrum: the composites retain the signal at 1606, 1416, 800, and 721 cm^−1^ from MOF‐525 and the signal at 690, 839, 981, 1089, and 1204 cm^−1^ from the PEDOT NTs, indicating that the MOF‐525 and PEDOT NTs coexist and that their individual functional groups and chemical structures are maintained in the composite.

**Figure 2:**
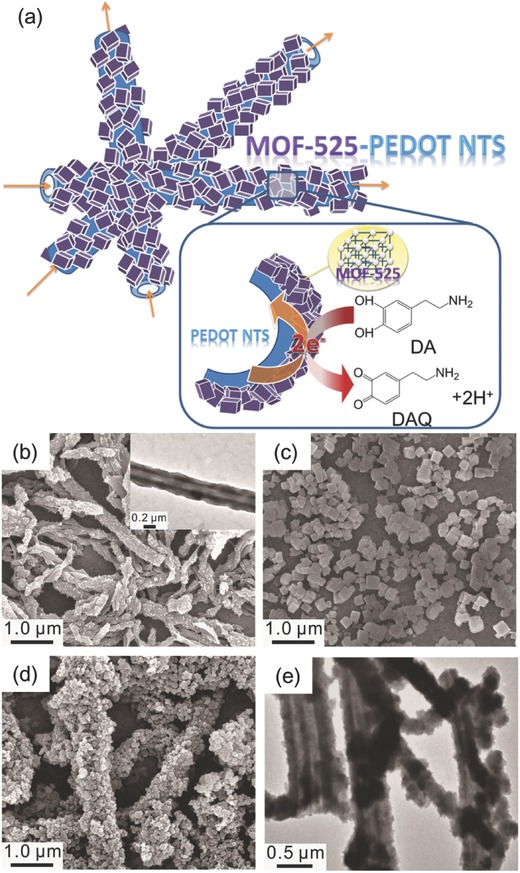
a) The scheme for the electrochemical detection of DA using MOF‐525–PEDOT NTs composite. SEM images of b) PEDOT NTs, c) MOF‐525 nanocrystals, and d) MOF‐525–PEDOT NTs. TEM images of e) MOF‐525–PEDOT NTs reveal that MOF‐525 coated onto PEDOT NTs, and the whole material can be connected to form an the interpenetrating network. The inset in (a) shows the TEM image for the hollow and smooth feature of one PEDOT NT.

The scheme in **Figure** **2**a shows that the MOF‐525 nanocrystals function as electrode materials with numerous electrochemically active sites, while the PEDOT NTs serve as charge collectors to efficiently transport electrons to the electrode. Such a MOF‐525–PEDOT NTs nanocomposite structure would provide synergistic effects that result in a marked improvement in conductivity and catalytic performance. Scanning electron microscopy (SEM) was first used to investigate the morphology of the pristine PEDOT NTs, MOF‐525, and the MOF‐525–PEDOT NT nanocomposite. As shown in Figure 2b, the surface of the PEDOT NTs was smooth, and the size of MOF‐525 nanocrystals is ≈500 nm (Figure 2c). In Figure 2d, the surface of PEDOT NTs is conformally coated with MOF‐525 nanocrystals. Solvothermal growth of MOF‐525 on the surface of PEDOT NTs enabled good contact for enhanced electron transport. Transmission electron microscopy (TEM) images in Figure 2e also support the conclusion that MOF‐525 nanocrystals decorate the surface of PEDOT NTs. Both SEM and TEM micrographs show that bundles of MOF‐525–PEDOT NTs formed an interconnecting network, which should help to improve the electrochemical properties of the film.

### 2.2 Electrochemical Impedance Analysis of MOF‐525–PEDOT NTs Composite Film

**Figure 3:**
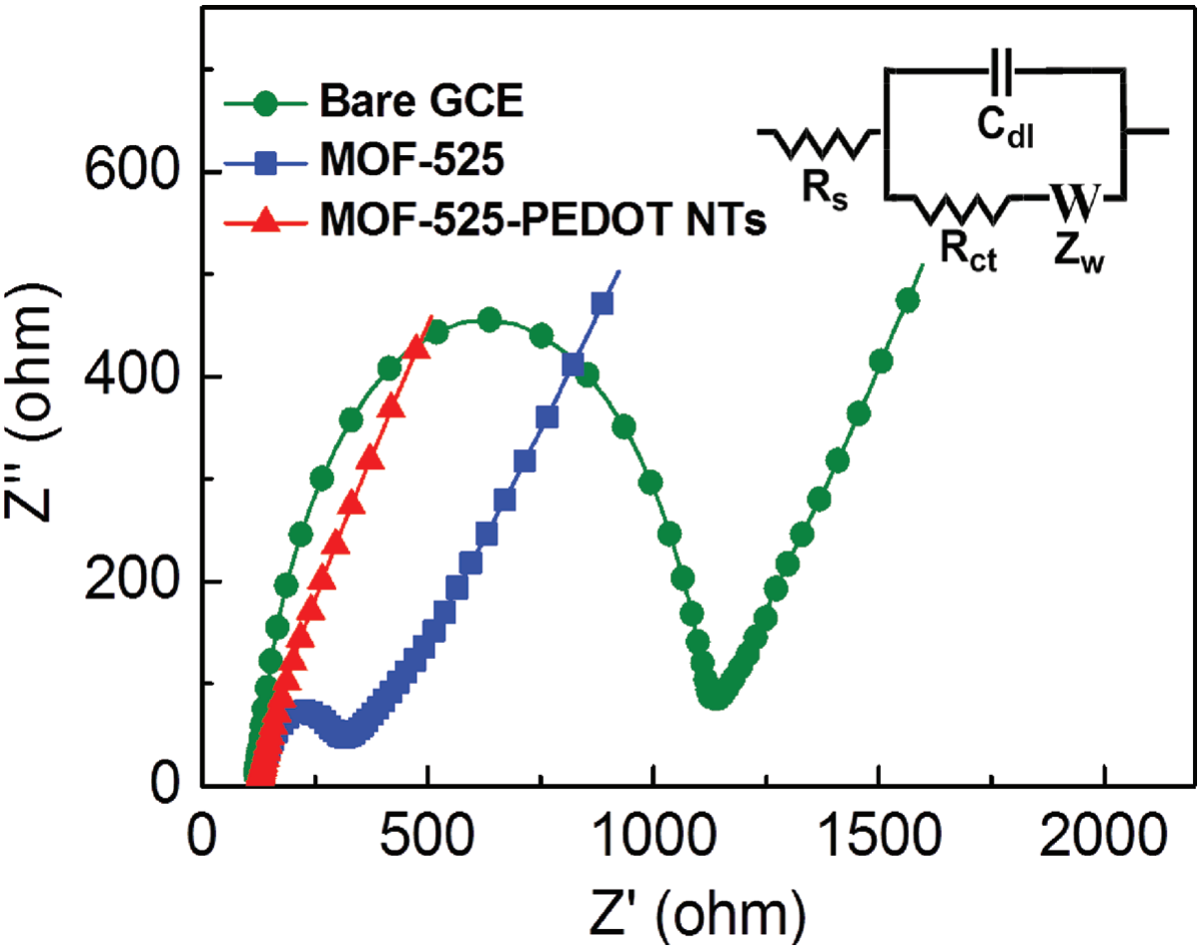
Nyquist plots of the bare GCE and GCE modified with MOF‐525, PEDOT NTs, and MOF‐525–PEDOT NTs tested in 0.1 m KCl solution containing 5 × 10^−3^ m Fe(CN)_6_^3−/4−^.

Electrochemical impedance spectroscopy (EIS) was utilized to study the charge resistance of the pristine MOF‐525 and MOF‐525–PEDOT NTs composite film. EIS can allow us to gain more insight into the morphological changes and electrochemical properties of the composite. The Nyquist plots of bare glassy carbon electrode (GCE), GCEs modified with MOF‐525 nanocrystals and MOF‐525–PEDOT NTs composite were studied in 0.1 m KCl solution containing 5 × 10^−3^ m Fe(CN)_6_^3−^/^4−^. As shown in the inset of **Figure** **3**, the *R*_s_ in the equivalent circuit is the series resistance for the overall system, while *Z*_w_ represents the resistance for ion diffusion in the electrolyte and *C*_dl_ describes the capacitive behavior of the film (Figure 3; inset). the *R*_ct_ represents the charge‐transfer resistance of the redox‐active film, which can be estimated from the region of the semicircle in Nyquist plot.^24^ Since charge is transported by redox hopping inside the MOF‐525 film, the charge transport rate is directly related to the charge‐transfer resistance of the film. The *R*_ct_ value of 1026.1 Ω for bare GCE decreased to 221.4 Ω after modifying with the redox‐active MOF‐525. Surprisingly, the *R*_ct_ value of the MOF‐525–PEDOT NT composite film decreased more than 20 times, to a value of 10.5 Ω. The markedly lower *R*_ct_ value indicates that inclusion of PEDOT minimizes the barrier of charge transport compared to the pristine MOF‐525 nanocrystal film. These results infer that MOF‐525–PEDOT NT nanocomposites not only facilitate the charge transport, but also accelerate the electrocatalysis for superior electrocatalytic activity. Besides, ultra‐high surface area of MOF‐525–PEDOT NT nanocomposites makes them efficiently to collect the charges for analyte detection.

### 2.3 Electrochemical Detection of Dopamine at MOF‐525–PEDOT NTs Electrode

**Figure 4:**
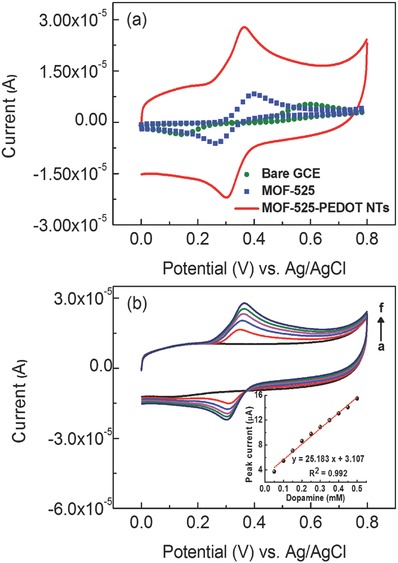
a) CV traces of the bare GCE and GCEs modified with MOF‐525, and MOF‐525–PEDOT NTs composite films in ABS containing 0.5 × 10^−3^ m DA; scan rate: 0.030 V s^−1^. b) CV curves of GCE modified with the MOF‐525–PEDOT NTs composite film with various concentrations of DA (0.05 × 10^−3^–0.5 × 10^−3^ m); scan rate: 0.030 V s^−1^.

CV was used to compare the electrocatalytic oxidation of DA (0.5 × 10^−3^ m) on the bare GCE versus GCEs modified with MOF‐525 and MOF‐525–PEDOT NTs composite films (**Figure** **4**a; Figure S1, Supporting Information). All CV traces showed a new redox peak that appeared after the addition of DA. As expected, the MOF‐525–PEDOT NTs composite film had the highest catalytic current toward the analyte. The oxidation peak current of the MOF‐525–PEDOT NTs composite electrode was 2.1 times higher than the MOF‐525 nanocrystal film, and 2.6 times higher than the pristine PEDOT NTs film. Moreover, the oxidation potential of the MOF‐525–PEDOT NTs composite film decreased slightly from 0.40 to 0.36 V compared to the MOF‐525 film. This was attributed to the reduced charge transport resistance of the film. In the electrocatalytic experiments shown in Figure 4b, there was an increase in the concentration of the analyte (from 0.05 × 10^−3^ to 0.5 × 10^−3^ m), resulting in a linear increase in the oxidation peak current of DA. These results indicate that the combination of MOF‐525 and PEDOT had a desirable effect on all aspects of DA sensing. An electrode composed of MOF‐525–PEDOT NTs can fully harness the advantage of numerous active sites from MOF materials via the improved charge transport imparted by the PEDOT NTs.

**Figure 5:**
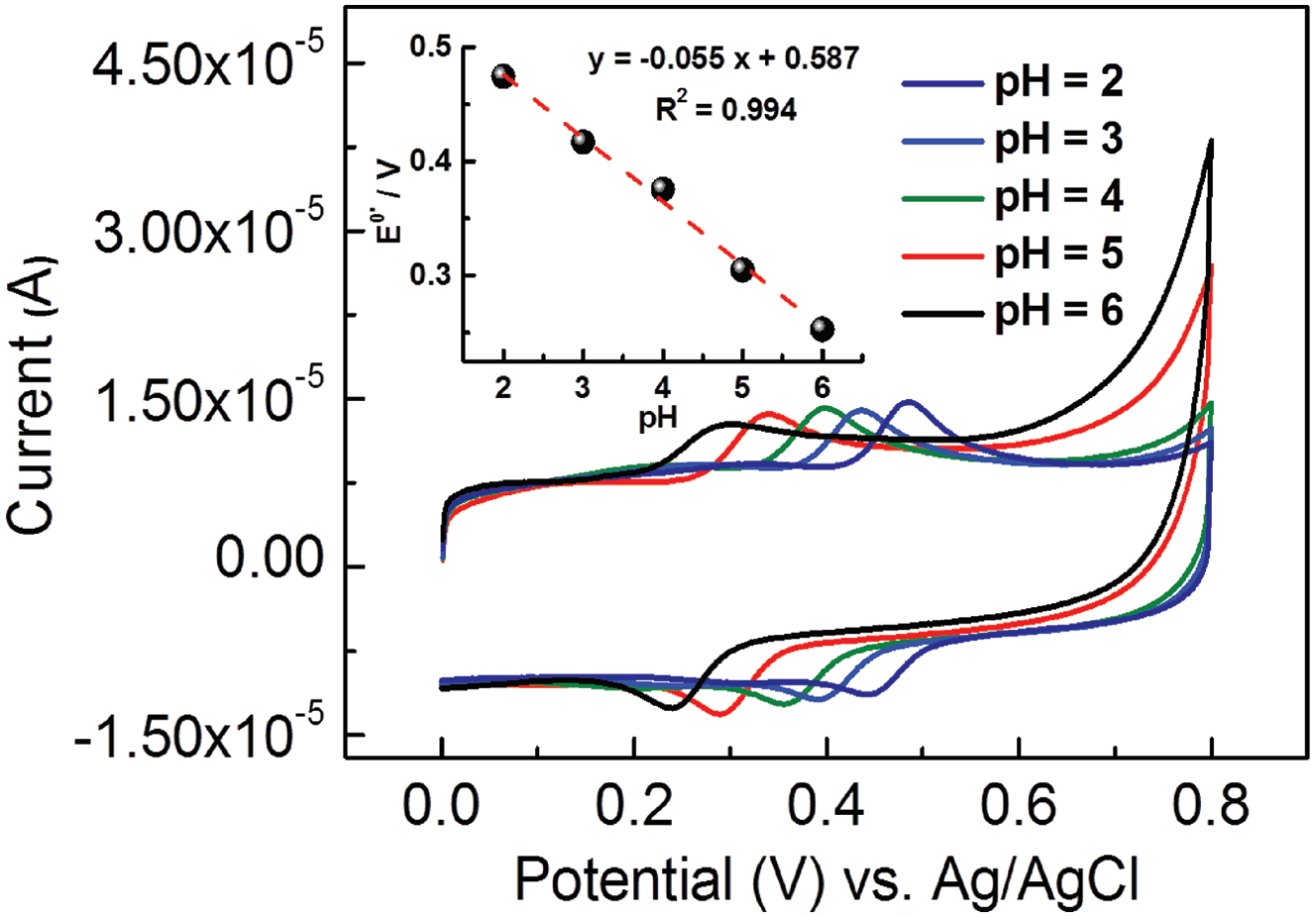
CV traces of the MOF‐525–PEDOT NTs modified electrode measured in the aqueous buffer solutions containing 0.1 × 10^−3^ m DA with various values of pH; scan rate: 0.030 V s^−1^. Inset: formal potential plotted with respect to the pH (2–6).

To further characterize the MOF‐525–PEDOT NTs modified electrode, the effect of pH was examined by CV. After recording the initial CV curve of the MOF‐525–PEDOT NT film in acetate buffer solutions (ABS), the sample and GCE were washed with deionized water and transferred to an aqueous buffer solution containing 0.1 × 10^−3^ m of DA at selected pH values (**Figure** **5**). The film was very stable between pH = 2 and pH = 6, with an obvious redox couple in each solution. In particular, the CV trace recorded under pH = 5 had a stable signal and a well‐defined redox couple, suggesting that the system at this pH would be ideal for analyte detection. Besides, to transfer the MOF‐525–PEDOT NT film in alkaline buffer solution, the redox couple in the CV traces dropped significantly, indicating that the MOF materials become unstable in alkaline conditions. The values of the peak potentials (Epa and Epc) were dependent on the pH of the buffer solution. The inset plots show the potential of the MOF‐525–PEDOT NT film versus pH. The slope of the response was 55 mV pH^−1^, which is essentially equivalent to theoretical value of 56 mV pH^−1^ from the Nernst equation for an equal number of proton and electron transfer processes.

**Figure 6:**
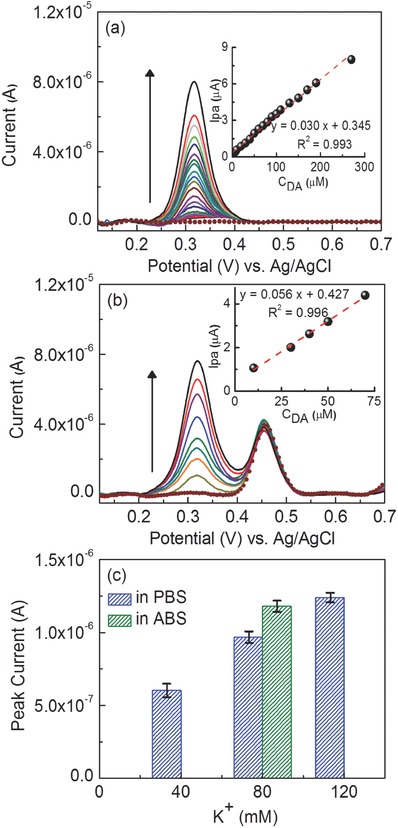
DPV curves of a) the GCE modified with MOF‐525–PEDOT NTs composite film measured in ABS (pH 5.0) with various concentrations of DA (2 × 10^−6^–270 × 10^−6^ m) and b) simultaneous detection of UA with varying concentrations of DA. Inset: linear dependence of the peak current with respect to the concentration of DA (10 × 10^−6^–70 × 10^−6^ m). c) The columns of plot indicate the catalytic response for DA released from living PC12 cells stimulating with different concentrations of K^+^ in PBS (0 × 10^−3^–120 × 10^−3^ m) and ABS (80 × 10^−3^ m).

The electrochemical detection of DA on the nanocomposite electrodes was further studied by the differential pulse voltammetry (DPV) technique. **Figure** **6**a shows the DPV traces of the MOF‐525–PEDOT NTs composite electrode in 0.1 m ABS (pH = 5) with various concentrations of DA. The catalytic peak current increased linearly with increasing concentration of DA. These MOF‐525–PEDOT NT films achieved a sensitivity of 0.428 µA μm ^−1^ cm^−2^ in the range of the DA concentration between 2 × 10^−6^ and 270 × 10^−6^ m (*R* ^2^ = 0.993) and the detection limit (S/N = 3) was estimated to be 0.04 × 10^−6^ m (Figure 6a; inset). The DPV response deviates from the calibration curve while in higher DA concentration, which could be attributed to the saturation of electroactive sites in nanocomposites. Additionally, ascorbic acid (AA) and uric acid (UA) were used to study how similar chemical analytes might interfere with DA sensing. DA, AA, and UA are highly electrochemically active, and their oxidation peaks are almost indistinguishable, making it difficult to develop an assay for instantaneous determination in electrochemical sensing applications. The MOF‐525–PEDOT NTs composite sample was examined in a solution containing DA, AA, and UA. In Figure 6b, the DPV traces of the nanocomposite electrode in 0.1 m ABS (pH = 5) contained the same concentration for AA and UA (100 × 10^−6^ m) with various concentrations of DA. In the separated experiment for the AA detection, the peak current is not observed after the addition of AA and there is also no oxidation current change for DA in the absence and presence of AA. Therefore, we could exclude possibility for the oxidation of DA due to presence of AA by catalytic (EC) reaction. The peak current for the addition of UA appeared at a potential between 0.4 and 0.5 V. Moreover, the signal for DA could be detected while the concentration of DA (10 × 10^−6^ m) is relative low to a concentration of AA and UA (100 × 10^−6^ m). The peak current from DA linearly increased with increasing concentration of DA. These results clearly demonstrate that the MOF‐525–PEDOT NTs composite electrode exhibited high sensitivity as well as excellent selectivity toward DA detection.

It is likely that the free‐base meso‐tetra(4‐carboxyphenyl)porphyrin (TCPP) of MOF‐525 plays an important role both in the electrocatalysis of DA and in the exclusion of any interference caused by AA and UA. Under the chemical conditions for sensing, cationic porphyrins (TCPP^+^) are readily formed in buffer solutions.^25^ Meanwhile, the amine group in DA is prone to form a stable ammonium ion owing to its electron lone pair. As the result, the primary amine group of DA in buffer solution can easily associate with the radical cationic TCPP^+^ and yield stable ammonium ion. For UA, the secondary amine group also exhibited similar affinity to TCPP^+^. However, the strong secondary amine group of UA shows less attraction to TCPP^+^ in buffer solution because it readily forms an ammonium ion with H^+^. The ammonium ion of UA electrostatically repels the TCPP^+^, leading to the very modest current response. The hydroxyl groups of AA displayed a weak affinity in buffer solution compared to DA and UA, resulting in a subtle current response. In other words, the MOF‐525 nanocrystals film facilitates the diffusion of DA to the electrode surface leading to superior electrocatalytic response compared to GCE, which is consistent with our CV results. The observation here is similar to the reported literature.^26^ Likewise, the mechanism also explains why the MOF‐525–PEDOT NTs modified electrode shows great specificity toward the DA detection.

We compared the MOF‐525–PEDOT NT sample with other DA sensors reported in the literature (Table S1, Supporting Information). Our sensor retains both a wide linear range (2 × 10^−6^–270 × 10^−6^ m) and the low detection limit (0.04 × 10^−6^ m). In the literature, although few modified electrodes showed low detection limits, their linear ranges were relatively narrow. For example, porphyrin‐functionalized graphene‐modified electrodes, gold@carbon dots‐chitosan modified electrodes, hollow nitrogen‐doped carbon microspheres modified electrodes, molecularly imprinted electropolymers/copper oxide nanoparticle‐modified electrodes, carbon‐functionalized metal organic framework/Nafion composites showed the lower detection limits of 0.01 × 10^−6^, 0.001 × 10^−6^, 0.02 × 10^−6^, 0.008 × 10^−6^ , and 0.008 × 10^−6^ m, respectively, toward the detection of DA, but their linear detection ranges were relatively narrow (0.01–70.0 × 10^−6^, 0.1–30 × 10^−6^, 5–70 × 10^−6^, 0.02–25 × 10^−6^, and 0.03–10 × 10^−6^ m, respectively).^27^ More importantly, the sensing performance of our MOF‐525–PEDOT NT sample was superior to several other carbon‐based materials, indicating that MOF–PEDOT nanocomposites are a good platform to expand the sensing capability to different analytes for electrochemical sensors.

### 2.4 Detection of Dopamine Release from PC12 Cells

We used live PC12 cells to assess the sensor by directly measuring DA released by the cells in solution. It has been reported that PC12 cells are stimulated by extracellular K^+^, leading to depolarization of the cell membrane, accompanied by the opening of voltage‐sensitive Na^2+^ and Ca^2+^ channel in that order.^28^ We used various concentrations of K^+^ solutions on the PC12 cells to depolarize them. While the opening of voltage‐sensitive Ca^2+^ channels leads to a gradual increase in the intracellular Ca^2+^ level, the cells will trigger exocytosis after reaching a sufficient threshold. Likewise, the increment of intracellular Ca^2+^ level prompts the release of DA from large vesicles of cells. Figure 6c shows the direct detection of DA released from PC12 cells. The CV response was recorded at various concentrations of K^+^ in phosphate buffer solution (PBS) solution (0 × 10^−3^–120 × 10^−3^ m). The current response of DA detection increased with increasing K^+^ by means of trigger the stronger exocytosis. To confirm the observed signals from the PC12 cells, a control experiment utilizing PC12 cells without K^+^ stimulation was performed and showed no obvious current response. Besides, the catalytic current of MOF‐525–PEDOT NTs composite electrode in ABS containing the concentration of K^+^ (80 × 10^−3^ m) is preferable, indicating that our nanocomposite exhibited highly sensing ability in ABS. The results with PC12 cells demonstrate that MOF‐525–PEDOT NTs composite films are useful for rapid and label‐free detection of biological exocytosis and could plausibly be implemented in a device to actively monitor DA levels in the human nervous system.

### 2.5 Stability and Reproducibility of MOF‐525–PEDOT NTs Electrode

Stability is a key challenge for practical sensing applications. We exposed the MOF‐525–PEDOT NT nanocomposite films to 100 continuous scan cycles in 0.1 m ABS at a scan rate of 100 mV s^−1^. After 100 scan cycles, the background current of the MOF‐525–PEDOT NTs modified electrode decreased by less than ≈5%. Furthermore, the sample maintained more than 90% of its original electrochemical activity toward DA after storing two weeks in air. To investigate reproducibility, five sensors fabricated separately under the same preparation were tested in the analyte solution. These samples showed the excellent reproducibility. In the interference test as shown in Figure S2 in the Supporting Information, the modified electrode also displays the good selectivity toward the analyte.

## 3 Conclusion

A novel nanocomposite composed of PEDOT NTs conformally coated with MOF‐525 nanocrystals was synthesized via in situ growth method. The MOF‐525–PEDOT NTs nanocomposites possess numerous electrochemical active sites because of the porphyrin unit in MOF‐525 nanocrystals. The one‐dimensional PEDOT NTs plays a critical role as a charge collector, enabling the transmission of the electron from the MOF‐525 nanocrystals. By combining the unique properties of PEDOT and MOF‐525, we obtained samples with enhanced electrochemical sensing performance. The MOF‐525–PEDOT NTs composites were used as DA sensors. This sample had a much higher catalytic current than the sum of its individual constituents and a good linear range of 2 × 10^−6^ –270 × 10^−6^ m with the detection limit of 0.04 × 10^−6^ m. The composite electrode also exhibited excellent selectivity for DA against common interferents and retains long‐term sensing ability. Furthermore, the direct detection of DA released from living PC12 cells demonstrates the practicality of the sensor as a device. Our findings clearly show that the MOF‐525–PEDOT NTs composite has great potential as a practical biosensor.

## 4 Experimental Section

*Chemicals*: TCPP (H_4_TCPP, Tokyo Chemical Industry Co., Ltd.), zirconyl chloride octahydrate (Alfa Aesar), *N*,*N*‐Dimethylformamide (DMF, Macron Fine Chemicals), benzoic acid and acetone (J. T. Baker), poly(3,4‐ethylenedioxythiophene) nanotubes (PEDOT NTs), DA, sodium nitrite (NO_2_^−^), potassium iodate (IO_3_^−^), L‐cysteine (L‐cys), AA, UA, hydrogen peroxide (H_2_O_2_), and methanol (MeOH) were purchased from Sigma–Aldrich. All other chemicals were of analytical grade and used without further purification. Aqueous solutions were prepared with doubly distilled water. The supporting electrolyte for the electrochemical studies was 0.1 m ABS (pH = 5), which was prepared from acetic acid and sodium acetate solutions. All solutions were deoxygenated by sparging with prepurified N_2_ gas.

*Apparatus and Measurement*: CV and DPV measurements were performed with a CHI 440 analytical system (CH Instruments). Each experiment used a conventional three‐electrode cell assembly, consisting of an Ag/AgCl reference electrode and a Pt wire counter electrode. The working electrode was either an unmodified GCE or a GCE treated with the sample (exposed area = 0.070 cm^2^). EIS measurements were performed with a potentiostat/galvanostat (Autolab, PGSTAT 30) equipped with FRA2 module. The GCEs measured in 0.1 m KCl solution containing 5 × 10^−3^ m Fe(CN)_6_^3−/4−^. All measurements were performed at 25 ± 2 °C. The surface morphology of the composite samples was observed with SEM (Nova NanoSEM 230). The MOF‐525–PEDOT NTs solution was dropped on a holey carbon‐coated copper grid (Lacey Carbon Type‐A 300 mesh copper grid, TED Pella) and then dried in vacuum overnight prior to characterization with TEM (JOEL JEM‐1230). X‐ray diffraction pattern analysis was conducted for phase identifications with a powder XRD (X'Pert PRO‐PANalytical, CuKa radiation). The IR spectra was measured by FT‐IR spectrometer (Perkin Elmer Spectrum 100).

*Preparation of MOF‐525–PEDOT NTs Modified Electrode*: The synthesis of pristine MOF‐525 nanocrystals had been described previously.^20^ To synthesize the MOF‐525–PEDOT NT nanocomposite particles, 1.35 g of benzoic acid and 105 mg of zirconyl chloride octahydrate were dissolved in 8 mL of DMF and placed into the gravity convection oven at 80 °C for 2 h. After cooling the solution to room temperature, 47 mg of H_4_TCPP and 25 mg of PEDOT NTs were added to this solution and then sonicated at room temperature for 20 min. Next, the solution was placed in an oven at 80 °C for 24 h to complete the reaction. The particles were collected by centrifugation and washed with DMF. The washing step was repeated two more times. Finally, the MOF‐525–PEDOT NT nanocomposite was suspended in DMF (5 mg mL^−1^) to prepare the modified GCE. Before starting each electrochemical experiment, GCEs were polished using a BAS polishing kit and a slurry of 0.05 μm aluminum oxide (Al_2_O_3_) powder and then rinsed sequentially with deionized water and ethanol. The GCEs were drop‐coated uniformly with MOF‐525–PEDOT NTs dispersion (2 µL) and dried in an oven. The obtained MOF‐525–PEDOT NT modified GCEs were washed carefully in deionized water to remove residual organic solvent and carbon and then dried at room temperature.

*Cell Culture and DA Detection from PC12 Cells*: The real‐time detection of DA release was carried out using living mammalian cells as a biological sample. In vitro experiments were carried out on a rat PC12 cell line, because of their wide use as a model for studying neurobiological functions and regulation of neurotransmitter release. PC12 cells were maintained in dulbecco's modified eagle's medium supplemented with 10% heat‐inactivated horse serum and 5% heat‐inactivated fetal bovine serum, 2 × 10^−3^ m l‐glutamine, 1% (v/v) Penicillin/Streptomycin and 1% sodium pyruvate. Cells were passaged every 5 days and incubated in a humidified, 37°C, 5% CO_2_ incubator. Prior to use, the culture dishes were coated with 0.01% poly‐l‐lysine and washed with PBS for three times. For the electrochemical experiments, PC12 cells were first detached by a mixture of trypsin‐EDTA, followed by centrifugation, and then suspended in regular culture medium. The cell suspension was diluted and aliquoted into a 24‐well plate. After 3 days, the culture medium was discarded, and the cells were washed three times with PBS. The cells were treated with 0 × 10^−3^, 40 × 10^−3^, 80 × 10^−3^, and 120 × 10^−3^ m KCl dissolved in 0.1 m PBS, 0 × 10^−3^ and 80 × 10^−3^ m KCl dissolved in 0.1 m ABS, and incubate in 37 °C, 5% CO_2_ incubator for 1 h.

## Conflict of Interest

The authors declare no conflict of interest.

## Supporting information

Supplementary
