# Supplementary material for: Enhanced Charge Collection in MOF‐525–PEDOT Nanotube Composites Enable Highly Sensitive Biosensing
Source: Adv Sci (Weinh). 2017 Sep 22;4(11):1700261. doi: 10.1002/advs.201700261 (PMC5700651; doi:10.1002/advs.201700261)
Supplement: Supplementary file 1 — Supplementary [file ADVS-4-na-s001.pdf]

Copyright WILEY-VCH Verlag GmbH & Co. KGaA, 69469 Weinheim, Germany, 2017.

ADVANCED SCIENCE
Open Access

Supporting Information

for *Adv. Sci.,* DOI: 10.1002/advs.201700261

Enhanced Charge Collection in MOF-525–PEDOT Nanotube Composites Enable Highly Sensitive Biosensing

Tzu-Yen Huang, Chung-Wei Kung, Yu-Te Liao, Sheng-Yuan Kao, Mingshan Cheng, Ting-Hsiang Chang, Joel Henzie, Hatem R. Alamri, Zeid A. Alothman, Yusuke Yamauchi, Kuo-Chuan Ho,* and Kevin C.-W. Wu**

Copyright WILEY-VCH Verlag GmbH & Co. KGaA, 69469 Weinheim, Germany, 2016.

Supporting Information

Enhanced Charge Collection in MOF-525-PEDOT Nanotube Composite Enable Highly Sensitive Biosensing

Tzu-Yen Huang, Chung-Wei Kung, Yu-Te Liao, Sheng-Yuan Kao, Mingshan Cheng, Ting-Hsiang Chang, Joel Henzie, Yusuke Yamauchi, Kuo-Chuan Ho* and Kevin C.-W. Wu**

Interference test

The amperometric response toward the addition of analyte was used to study the electrocatalytic oxidation of DA by the MOF-525-PEDOT NT nanocomposite electrode. The applied potential was set at 0.33 V with an electrode rotation speed of 1200 rpm. In **Figure S2**, the amperometric response of the MOF-525-PEDOT NT film was measured as a blank for the initial period of 100 s. After injecting 0.5 mM DA at 100 s, various interferents including 1.0 mM NO^{2-}, 1.0 mM IO^{3-}, 1.0 mM L-cys, 1.0 mM AA, 1.0 mM H_2O_2 and 1.0 mM MeOH, were successively injected into the buffer solution. The current response for all interferents was less than 5%. It is also important to note that DA was injected to the solution after all additions of those interferents. The MOF-525-PEDOT NT film still showed a high catalytic selectivity toward DA. The results show that our sample has excellent sensing ability for DA in the presence of interferents and still had high selectivity toward the analyte.

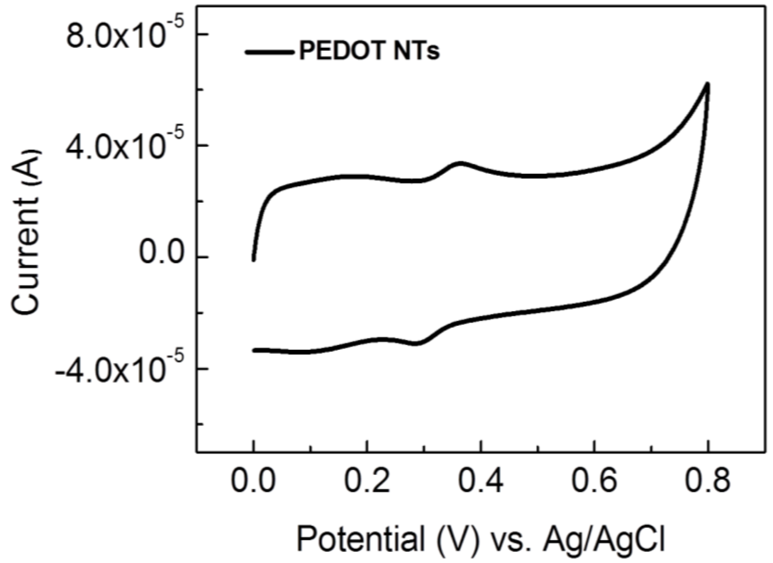

Figure S1. CV trace of the GCE modified with PEDOT NTs film in ABS containing 0.5 mM dopamine; scan rate: 30 mV s^{-1}

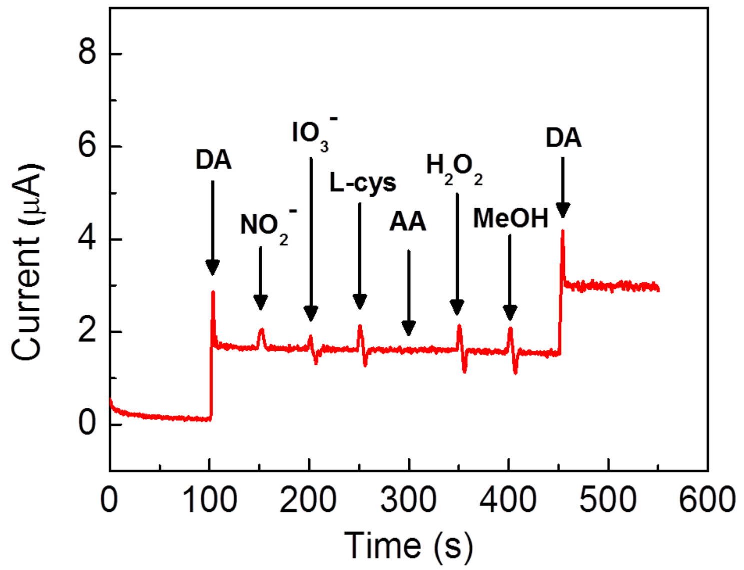

Figure S2. Interference test of the amperometric response of the MOF525–PEDOT NTs composite film in stirred ABS, with the injections of NO^{2-}, IO^{3-}, L-cys, AA, H_2O_2 and MeOH successively.

Table S1. Comparison of dopamine detection for the reported electrochemical sensors

Modified electrodes	Limit of detection (µM)	Linear range (µM)	pH	Ref.
Porphyrin-functionalized graphene	0.01	0.01-70	7.0	[1]
Au@carbondots–chitosan	0.001	0.1-30	7.0	[2]
Graphene	2.64	4.0-100	7.4	[3]
Hollow nitrogen-doped carbon microspheres	0.02	5-70	7.0	[4] _ENREF_56
Molecularly imprinted electropolymers @CuO	0.008	0.02-25	7.5	[5] _ENREF_57
Au-nanoclusters incorporated 3-amino-5-mercapto-1,2,4-triazole	0.05	0.6-340	4.0	[6]
Polyimidazole/graphene oxide copolymer	0.63	12-278	3.0	[7]
Carbon functionalized metal organic framework/Nafion composites	0.008	0.03-10	5.0	[8]
SiO_2-coated graphene oxide	0.03	0.05-160	7.0	[9]
Graphene/poly-cyclodextrin/MWCNT	0.05	0.15-21.65	6.0	[10]
Copper terephthalate metal−organic framework−graphene oxide	0.21	1-50	5.0	[11]
MOF-525−PEDOT NTs	0.04	2-270	5.0	This work

References

[1] L. Wu, L. Feng, J. Ren, X. Qu, *Biosens. Bioelectron.* 2012, 34, 57.

[2] Q. Huang, H. Zhang, S. Hu, F. Li, W. Weng, J. Chen, Q. Wang, Y. He, W. Zhang, X. Bao, *Biosens. Bioelectron.* 2014, 52, 277.

[3] Y.-R. Kim, S. Bong, Y.-J. Kang, Y. Yang, R. K. Mahajan, J. S. Kim, H. Kim, *Biosens. Bioelectron.* 2010, 25, 2366.

[4] C. Xiao, X. Chu, Y. Yang, X. Li, X. Zhang, J. Chen, *Biosens. Bioelectron.* 2011, 26, 2934.

[5] B. Li, Y. Zhou, W. Wu, M. Liu, S. Mei, Y. Zhou, T. Jing, *Biosens. Bioelectron.* 2015, 67, 121.

[6] C. Wang, R. Yuan, Y. Chai, Y. Zhang, F. Hu, M. Zhang, *Biosens. Bioelectron.* 2011, 30, 315.

[7] X. Liu, L. Zhang, S. Wei, S. Chen, X. Ou, Q. Lu, *Biosens. Bioelectron.* 2014, 57, 232.

[8] Y. Wang, H. Ge, G. Ye, H. Chen, X. Hu, *J. Mater. Chem. B* 2015, 3, 3747.

[9] Y. Zeng, Y. Zhou, L. Kong, T. Zhou, G. Shi, *Biosens. Bioelectron.* 2013, 45, 25.

[10] Y. Zhang, R. Yuan, Y. Chai, W. Li, X. Zhong, H. Zhong, *Biosens. Bioelectron.* 2011, 26, 3977.

[11] X. Wang, Q. Wang, Q. Wang, F. Gao, F. Gao, Y. Yang, H. Guo, *ACS Appl. Mater. Interfaces* 2014, 6, 11573.
